# Place of death for people with dementia: A scoping review

**DOI:** 10.1371/journal.pone.0340024

**Published:** 2026-01-09

**Authors:** Paula Hidalgo-Andrade, Alejandro Unda-López, Tania Pastrana

**Affiliations:** 1 Grupo de investigación Bienestar, Salud y Sociedad, Universidad de Las Américas, Campus UDLAPARK, Quito, Ecuador; 2 Department of Palliative Medicine, Medical Faculty, RWTH Aachen University, Germany; 3 International Association for Hospice and Palliative Care (IAHPC), Houston, Texas, USA; University Hospital Cologne: Uniklinik Koln, GERMANY

## Abstract

**Background:**

Dementia is a global health concern that contributes significantly to severe health-related suffering and poses high demands on healthcare systems, patients, families, and caregivers. Caring for people with dementia should include all the stages of illness, including end-of-life care.

**Aim:**

Given that the place of death is a crucial aspect of end-of-life care, we aimed to analyze the scientific literature on the place of death of individuals with dementia, the sociodemographic and contextual variables associated with it, and evidence-based suggestions to improve end-of-life care for people with dementia.

**Methods:**

We conducted a scoping review in 19 databases in November 2023 and updated the search in February 2025, yielding 1590 and 660 sources of evidence, respectively. Manuscripts had to a) focus on the frequency of more than one place of death, b) include population with dementia, c) use administrative data, and d) be published in peer-reviewed primary sources to be included in this study. The procedure for searching the sources of evidence and data charting was based on the structured approach for conducting scoping reviews by Arksey and O’Malley. Pre-registration can be retrieved at https://doi.org/10.17605/OSF.IO/BVXMY.

**Results:**

After screening, a total of 48 studies were included in this review. Results indicate a great variability in the place of death of people with dementia across different regions and settings. Overall, in Europe and North America, it was more common for people with dementia to die in long-term care facilities like care homes, nursing homes, and sheltered housing; in East Asia and the Pacific, medical facilities (i.e., hospitals, mental health facilities, and private clinics) were more common. Studies that showed trends also indicate an increase in long-term-care facilities and a decrease in medical facilities and homes as the place of death. Most studies were conducted in high-income countries, raising questions about the diverse factors that may influence the place of death, such as access to services and socioeconomic status.

**Discussion:**

These results could inform decisions on allocating resources and training for end-of-life care for people with dementia. However, more research is needed to increase the representativeness of other world regions and understand other variables that influence the place of death.

## Introduction

Dementia is a progressive neurodegenerative noncommunicable disease that affects over 55 million people worldwide [1]. Its prevalence has increased primarily due to population growth and extended life expectancies resulting from advancements in healthcare [2,3]. It is a global health concern, ranking among the leading causes of care dependency and disability [3], significantly contributing to severe health-related suffering [4,5], and increasing the demands on healthcare systems and families or caregivers [6]. These characteristics make dementia care particularly complex.

As dementia advances to severe stages, individuals experience significant impairments in memory, judgment, and decision-making capacity, often rendering them unable to express their symptoms, preferences, or discomfort [7]. Additionally, people with dementia (PwD) often suffer from multiple additional diseases or comorbidities [8], including infections, delirium, aspiration pneumonia, among others. These conditions require an individualized and sensitive approach to care that balances symptom management, psychological support, and ethical considerations such as autonomy and dignity [9]. The needs of PwD and their families also include end-of-life care and the alignment between preferred and actual place of death (PoD) [10]. Ideally, PwD and their families should have the autonomy to choose where they die, with every location providing high-quality end-of-life care characterized by adequate symptom management and access to treatments [10,11]. The preferred PoD is used as a quality indicator for end-of-life care [12]. Studying PoD allows healthcare providers and policymakers to better understand, plan, and improve the care of PwD as well as to address the issues surrounding death.

Research indicates variability in PoD of PwD among countries, potentially linked to differences in the organization of end-of-life care provision [13]. The World Health Organization recommends that dementia care be integrated into national health plans, policies, and legislation to enhance support for PwD and their families, while also alleviating the burden on health systems [3]. Relevant information is needed to make informed decisions and adhere to those recommendations. However, the complexity of caring for PwD and various other factors may influence the place of care and PoD. A recent study showed that 10 factors influence the PoD of individuals with advanced dementia: age, gender, ethnicity, presence of pneumonia, chronic obstructive pulmonary disease, cancer, functional status, marital status, urbanization level, and funding [14].

Cultural and social factors have also been further studied in relation to PoD. The interplay of cultural values, healthcare infrastructure, familial and community support networks, and cultural attitudes toward caregiving can influence where individuals with dementia die. In Taiwan, for example, a study showed that the high prevalence of home deaths (not only of PwD) has been partly attributed to the practice of “impending death discharge”, wherein terminally ill individuals are released from hospitals to die at home following Chinese cultural traditions [15].

A recently published meta-analysis conducted by Tay and colleagues [14] offers a rigorous quantitative synthesis of evidence up to 2022 and focuses narrowly on the factors associated with PoD in people with advanced dementia. Our scoping review addresses broader research questions and incorporates diverse study designs, populations, and outcomes to offer current, contextual insights essential for understanding the PoD of PwD in recent evidence. Specifically, this scoping review expands on this by addressing three broader questions:

1) Where do PwD die?
2) Which sociodemographic and contextual variables explain variations in the PoD of PwD?
3) What evidence-based suggestions can be drawn from the research to improve end-of-life care provision for PwD?

## Methods

The present scoping follows the PRISMA extension for scoping reviews (PRISMA-ScR) guidelines [16] (Supplementary Material 1).

### Protocol and registration

We conducted a scoping review, ideal for identifying the scope or coverage within an existing body of literature and providing an overview of a studied topic [17]. This review pre-registration is available on the Open Science Framework (OSF) platform (https://doi.org/10.17605/OSF.IO/BVXMY). The final study had two deviations from the study registration. First, we did not address the question of PoD preferences because, after data extraction, we noticed that information on this subject was missing. As no results were drawn, the question was not addressed. Second, the planned quality assessment was not conducted because, as scoping reviews’ objectives center on mapping the existing evidence, no quality appraisal was deemed necessary. The procedure for searching the sources of evidence and data charting was based on the six-stage framework by Arksey and O’Malley [18]. The primary and secondary research questions were established for the first stage. For the second stage, databases were selected to identify and retrieve relevant studies based on their affinity with the topic studied and the coverage of evidence sources.

### Eligibility criteria

To be included in the review, manuscripts had to meet all the following inclusion criteria: 1) Focus on the distribution of more than one PoD of people with dementia. 2) be a peer-reviewed published manuscript; and 3) use administrative data (information gathered and maintained by government agencies or official organizations for administrative purposes). Articles that focused solely on pre-senile dementia were excluded from the present review, as the term and diagnosis are outdated. This term was used to frame cognitive impairments, especially memory loss, as a natural consequence of ageing rather than the result of a disorder [19,20], and has been used ambiguously to refer either to symptomatology associated with Alzheimer’s disease or non-specific losses in cognitive function [20]. As these diagnoses are inconsistently applied through the literature, such populations may not be representative of typical patients dying with dementia, and end-of-life care considerations may not be comparable. No study design, date limit, or language was considered as a criterion. For articles in languages not spoken by the authors, AI tools were implemented for translation.

### Information sources

**Fig 1:**
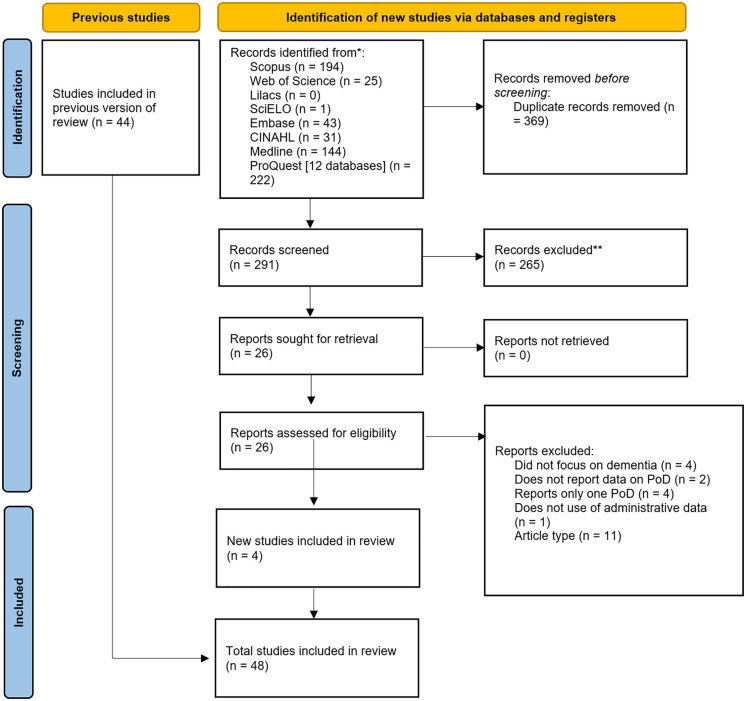
PRISMA 2020 flow diagram for updated searches [26]. *Databases [12] accessed through the ProQuest interface were PsycINFO (APA), PRISMA Database (1966—current), Biological Science Database, Health & Medical Collection, Healthcare Administration Database, Latin America & Iberia Database, Nursing & Allied Health Database, Psychology Database, Public Health Database, Publicly Available Content Database, Social Science Database, and Sociology Database (1985—current).

A search was conducted on 19 databases (see Fig 1) from November 4 to November 9, 2023, to identify publications. A second search was conducted between February 9 and February 12, 2025, to update the sources of evidence included.

### Search

The search string contains terms related to dementia and PoD (refer to pre-registration for specific queries in each database). An example of the query used (for Scopus) can be seen next:

**Query string:** TITLE-ABS-KEY(dementia OR alzheimer’s OR alzheimer OR “alzheimer’s disease” OR “senile dementia” OR demented) AND TITLE-ABS-KEY(“place of death” OR “death place” OR “site of death” OR “location of death” OR “home death” OR “hospital death” OR “inpatient death” OR “nursing home death” OR “hospice death” OR “aged care home death” OR “residential care home death”).

### Selection of sources of evidence

In the third stage, we conducted an initial review, excluding articles that did not meet the inclusion criteria. Sysrev (http://www.sysrev.com), a collaborative working platform useful in several stages of conducting reviews, was used for data management and screening. Two researchers (PHA, AUL) conducted the screening in three steps: 1) Duplicates were removed, 2) two reviewers independently screened titles and abstracts against inclusion criteria, and 3) full texts were retrieved and assessed independently for final inclusion. Discrepancies between all researchers were discussed after steps two and three.

### Data charting process

For the fourth stage, data were charted using an online form created for this study. Three researchers (PHA, TP, AUL) conducted data extraction independently, and discrepancies were discussed in team meetings.

### Data items

To describe the characteristics of the included studies, data from journal details (name and journal region), manuscript information (title, year of publication, and language), and research information (aim, study design, and context) were extracted.

The following data were charted to answer the present research questions. For the first research question, items aimed at attaining data on sample characteristics, study location(s), participants’ country of origin, and significant results on the PoD. To approach the second question, data regarding sociodemographic characteristics, type of dementia, and factors associated with PoD were extracted. And for the third question, charted data focused on reported conclusions, limitations, and suggestions or recommendations for theory and practice.

### Critical appraisal

There is an ongoing debate on whether quality appraisal is part of a scoping review process [21], although it is suggested that not conducting this step may be presented as a limitation in this methodology [18]. The present review aimed to review peer-reviewed papers with non-experimental designs using administrative data. As scoping reviews provide an overview of the evidence within a given topic [22], quality appraisal was not conducted to avoid further limiting the inclusion of potentially relevant studies.

### Synthesis of results

In the fifth stage, data obtained from included articles were collated and summarized using a spreadsheet. An inductive approach was used for identifying trends in the data. All team members were involved in this stage. Disagreements were resolved during team meetings. Synthesis of results was presented based on the PRISMA-ScR guidelines and followed the same order as the research questions presented above. Additionally, we performed some data transformations. First, when information on the frequency/percentage of PoD was not directly available, we calculated it whenever possible. Second, given the heterogeneity in PoD reports, we reclassified them into the following categories: ‘Home’, ‘Medical facilities’ (MF), ‘Long-term care facilities’ (LTCF), ‘Palliative care facilities’ (PCF), and ‘Elsewhere’. Table 1 presents the specific locations reported by the included studies and their categorization within each new category.

**Table 1:** Categorization of PoD.

Category	PoD included in each category
**Home**	Private address; Home; Residential Care (Homeowner within a fully equipped community); Residential Home (Facility for living under care or supervision).
**Medical facilities (MF)**	Acute Hospital, Cottage Hospital, District General Hospital, Hospital, Intensive Care Unit, Intermediate Geriatric Care Facility, Long-stay Hospital, Mental Health Facility, Private Clinic, Psychiatric Hospital, Specialized Care Hospital.
**Long-term care facilities (LTC)**	Care Home, Nursing Home, and Sheltered housing.
**Palliative care facilities (PCF)**	Hospice, Palliative Care Unit (no hospital)^a^.
**Elsewhere** ^**b**^	Elsewhere, Other, Unknown.

**Note:** ^a‘^Palliative care unit’ is characterized as having a specialized focus on palliative care [23–25]; therefore, it was categorized as PCF and not as MF.

^b^The category ‘Elsewhere’ contains the places of death that studies deemed as not belonging to the previously established categories (e.g., on the street) or as unknown.

## Results

### Selection of sources of evidence

The first search yielded a total of 1590 manuscripts, of which 963 were duplicates. 627 records were screened in the first stage, and 112 manuscripts were assessed for eligibility. A total of 44 studies reached the charting stage. For the second search conducted, a total of 660 manuscripts were identified, 26 were assessed for eligibility, and 4 studies reached the final stage. Fig 1 shows the selection of sources of evidence for the updated search.

### Sample characteristics

After the selection process, 48 articles were included (see Fig 1), with samples ranging from 147 [27] to 3,291,422 [28]. The overall age ranged from 41 to 110 years; additionally, studies focused on male and female participants alike, except for a study that only included male participants with dementia [29].

Data on dementia-related diagnoses were collected to ensure comparability of the studies. Thirty-one studies reported using diagnoses from the International Statistical Classification of Diseases and Related Health Problems 10^th^ Revision (ICD-10), including vascular dementia (F01), Dementia in other diseases classified elsewhere (F02), unspecified dementia (F03), Alzheimer’s disease (G30), other degenerative diseases of nervous ystem, not elsewhere classified (G31), other degenerative disorders of nervous system in diseases classified elsewhere (G32), senility (R54), and their subtypes [13,23–25,28–54]. Additionally, one article included diagnoses from both International Statistical Classification of Diseases and Related Health Problems 9^th^ Revision (ICD-9) involving dementias (code 290), persistent mental disorders due to conditions classified elsewhere (code 294), and other cerebral degenerations (code 331) and the ICD-10 diagnosis F01, F02, F03, G30, and G31 [55].

Six articles reported diagnoses without a specific classification, including Alzheimer’s disease and dementia [56–61], senility [57,58], vascular dementia [56,60,61], unspecified dementia [56,61], and Lewy-body dementia [61]. Two studies reported Mini-Mental State Examination (MMSE) scores for determining the severity of dementia [27,62]. Finally, eight studies reported dementia as a characteristic of the sample without further specification [63–70].

### Study characteristics

Out of the 48 included articles, 44 (91.7%) were published in English, three (6.3%) in German [25,34,63], and one (2%) in Japanese [56]. The articles were published between 1997 and 2024. All used a non-experimental design, of which 41 (85.4%) reported a retrospective approach with a single data collection time, five (10.4%) reported a time series design, and two (4.2%) reported a cross-sectional design with a prospective approach. For data attainability, 25 (52.1%) studies used death certificates, with one study (2.1%) using obituaries as complementary sources of information [64]. Additionally, six other studies (12.5%) used death registers as data collection tools, and 17 (35.4%) used mortality statistics. Additionally, 26 studies (54.2%) included data from 12 European countries; nine studies (18.8%) included data from East Asia and the Pacific, specifically New Zealand, Japan, and the Republic of Korea; on the other hand, fourteen studies (29.2%) included data from North America, specifically from the United States and Canada; and one study (2.1%) included data from Latin America and the Caribbean, delving on Mexican PwD. Notably, no study included participants from Africa, Central and South Asia, or the Middle East.

### The place of death of people with dementia

Findings show that LTCF are the most frequent PoD in Belgium [13,37,38], Czech Republic [13], England [13,27,37,51,61], Germany [24,25,34,63], Northern Ireland [70], Norway [35,45,65], Scotland [37], Sweden [47], and the Netherlands [13,37,44,46,67].

Conversely, nine studies (18.8%) conducted in the same region reported MF as the most common PoD [25,27,30,31,57,60,62]. Moreover, two multicentric studies (4.2%) found the same trend for Wales/UK [38], France, and Hungary [13]. In addition, a few studies found that Home was the most common PoD in Scotland/UK [23], Portugal [58], Italy, and the Andalusian region in Spain [13]. Finally, two studies (4.2%) found similar proportions for MF and LTCF in Belgium with 47% for each when pneumonia was present as a comorbidity [38] in Finland, with 46.7% for MF and 45.2% for LTCF [48], and Wales where a study set in 2003 found 52.8% of deaths occurred in MF and 43.8% in LTCF [37], while a study including deaths from 2011 to 2013 found a 43.1% of death happened in MF and 48.9% in LTCF [13].

For East Asia and the Pacific, seven studies (14.6%) reported MF as the most common PoD in this region [13,29,36,42,50,52,53,56]. However, two studies (4.2%) reported a higher proportion of deaths in LTCF for PwD in New Zealand [13] and Japan [43].

In North America, MF was reported as the most frequent PoD in Canada and the United States [13,32,33,54,55,59,66,69]. In addition, four studies (8.3%) reported LTCF as the most prevalent [28,39,40,64], while two (4.2%) identified home as the most common PoD [41,68]. Notably, only one (2.1%) study included people from Latin America and the Caribbean in their data [13], reporting home as the most frequent PoD in Mexico. No study reported PCF as the most frequent PoD. Table 2 shows the distribution of PoD proportions in detail.

**Table 2:** Frequency of place of death of people with dementia in each country.

Study and sample size^c^	Year of death	Countries	Context	Comorbidities or other reported characteristics^b^	Place of death (%)^a,b^				
					Home	Medical Facilities	Long-Term Care Facilities	Palliative Care Facilities	Elsewhere
**Europe (26 studies)**									
Houttekier et al., 2010	2003	Belgium	Country	N/A	16.4	22.8	60.6	0.1	N/A
n = 30281									
Houttekier et al., 2013	2008	Belgium	Country	Without-pneumonia	11	28	61	N/A	N/A
n = 9931									
Houttekier et al., 2013	2008	Belgium	Country	With-pneumonia	6	47	47	N/A	N/A
n = 9931									
Reyniers et al., 2015	2011-2013	Belgium	Country	N/A	11.2	21.6	66.7	N/A	0.5
n = 264604									
Reyniers et al., 2015	2011-2013	Czech Republic	Country	N/A	10.6	27.5	61.5	N/A	0.4
n = 264604									
Perrels et al., 2014	1985-2007	England, UK	City: Cambridge city	Moderate cognitive impairment	45	55	0	N/A	N/A
n = 168									
Perrels et al., 2014	1985-2007	England, UK	City: Cambridge city	Severe cognitive impairment	42	46	13	N/A	N/A
n = 168									
Fleming et al., 2017	1985-2015	England, UK	City: Cambridge city	Minimal-mild dementia	15	62	23	N/A	N/A
n = 147									
Fleming et al., 2017	1985-2015	England, UK	City: Cambridge city	Moderate-severe dementia	5	20	76	N/A	N/A
n = 147									
Sleeman et al., 2014	2001-2010	England, UK	Country	N/A	4.8	39.58	55.33	0.29	N/A
n = 397573									
Houttekier et al., 2010	2003	England, UK	Country	N/A	4.9	39.1	55.4	0.2	N/A
n = 30281									
Reyniers et al., 2015	2011-2013	England, UK	Country	N/A	5	31.7	61.7	0.1	1.5
n = 264604									
Wiggins et al., 2019	2015-2017	England, UK	City: London	N/A	26.5	12.1	50.9	3.2	7
n = 1047									
Masuchi et al., 2017	1998-2013	Finland	Country	N/A	8.1	46.7	45.2	N/A	N/A
n = 140034									
Brosselin et al., 2010	2000–2006	France	Country	N/A	24.5	43.8	29.1	N/A	2.6
n = 45597									
Reyniers et al., 2015	2011-2013	France	Country	N/A	27.2	35.9	34	N/A	2.9
n = 264604									
Dasch & Lenz, 2022	2001	Germany	Region: Westphalia	N/A	24	40.4	35.2	0.4	N/A
n = 420									
Dasch et al., 2018	2011	Germany	Region: Westphalia–Lippe	N/A	19.87	28.74	49.51	1.21	0.67
n = 1646									
Dasch & Lenz, 2022	2011	Germany	Region: Westphalia	N/A	19.7	29	49.5	1.2	N/A
n = 420									
Dasch & Lenz, 2021	2017	Germany	City: Munster	N/A	12	23.1	63.7	1.2	N/A
n = 485									
Dasch & Lenz, 2022	2017	Germany	Region: Westphalia	N/A	15.8	24.3	57.1	2.7	N/A
n = 420									
Dasch, 2023	2021	Germany	City: Munster	N/A	14.45	25.41	58.62	1.52	N/A
n = 1578									
Reyniers et al., 2015	2011-2013	Hungary	Country	N/A	N/A	62.3	N/A	N/A	37.7
n = 264604									
Reyniers et al., 2015	2011-2013	Italy	Country	N/A	42.2	32.1	19.5	N/A	6.2
n = 264604									
Basso et al., 2023	2012-2015	Italy	Region: Veneto	MCOD^e^	20.7	42.3	33.2	N/A	3.7
n = 70301									
Basso et al., 2023	2012-2015	Italy	Region: Veneto	UCOD^d^	20.2	39.6	36.9	N/A	3.3
n = 70301									
Basso et al., 2023	2016-2019	Italy	Region: Veneto	MCOD	19.4	40.6	36.2	N/A	3.8
n = 70301									
Basso et al., 2023	2016-2019	Italy	Region: Veneto	UCOD	19.4	38.3	38.6	N/A	3.6
n = 70301									
Basso et al., 2023	2020	Italy	Region: Veneto	MCOD	19.6	37.1	40.5	N/A	2.8
n = 70301									
Basso et al., 2023	2020	Italy	Region: Veneto	UCOD	21.1	34.2	42.3	N/A	2.5
n = 70301									
Zafeiridi et al., 2021	2010-2016	Northern Ireland, UK	Country	N/A	N/A	N/A	64.35	35.65	N/A
n = 2581									
MacNeil-Vroomen et al., 2020	2006	Norway	Country	N/A	4.4	9	84.9	N/A	1.6
n = 61940									
MacNeil-Vroomen et al., 2020	2007	Norway	Country	N/A	5.1	8.7	85.9	N/A	1
n = 61940									
MacNeil-Vroomen et al., 2020	2008	Norway	Country	N/A	4.7	9.4	84.6	N/A	1.2
n = 61940									
MacNeil-Vroomen et al., 2020	2009	Norway	Country	N/A	4.4	7.5	86.7	N/A	1.5
n = 61940									
MacNeil-Vroomen et al., 2020	2010	Norway	Country	N/A	4.5	7.9	86.8	N/A	0.7
n = 61940									
MacNeil-Vroomen et al., 2020	2011	Norway	Country	N/A	4.3	6.7	87.5	N/A	1.5
n = 61940									
Hagen & Zelko, 2024	2011-2013	Norway	Country	N/A	15.6	N/A	84.4	N/A	N/A
n = 2900									
MacNeil-Vroomen et al., 2020	2012	Norway	Country	N/A	5.1	6.9	86.9	N/A	1.1
n = 61940									
Kjellstadli et al., 2018	2012-2013	Norway	Country	N/A	5.03	3.87	90.9	0.2	N/A
n = 5447									
MacNeil-Vroomen et al., 2020	2013	Norway	Country	N/A	4.8	6.7	87.1	N/A	1.5
n = 61940									
MacNeil-Vroomen et al., 2020	2014	Norway	Country	N/A	5.4	6.8	86.6	N/A	1.2
n = 61940									
MacNeil-Vroomen et al., 2020	2015	Norway	Country	N/A	4.9	7	87	N/A	1.1
n = 61940									
MacNeil-Vroomen et al., 2020	2016	Norway	Country	N/A	2.8	6.9	98.1	N/A	1.3
n = 61940									
MacNeil-Vroomen et al., 2020	2017	Norway	Country	N/A	2.6	6	90.5	N/A	0.9
n = 61940									
Gomes et al., 2018	2010-2016	Portugal	Country	N/A	80.5	19.5	N/A	N/A	N/A
n = 40880									
Thomas et al., 1997	1974-1994	Scotland, UK	Country	Vascular dementia	13.79	86.21	N/A	N/A	N/A
n = 1492									
Thomas et al., 1997	1974-1994	Scotland, UK	Country	Alzheimer’s disease	7.79	92.21	N/A	N/A	N/A
n = 1492									
Black et al., 2016	2000–2010	Scotland, UK	Region: Dumfries and Galloway	N/A	81.2	18.2	N/A	0.6	N/A
n = 545									
Houttekier et al., 2010	2003	Scotland, UK	Country	N/A	7.3	37.6	54.9	0.3	N/A
n = 30281									
Reyniers et al., 2015	2011-2013	Spain	Region:	N/A	46.1	33.6	20.1	N/A	0.2
n = 264604			Andalusia						
Cabañero-Martínez et al., 2019	2012-2015	Spain	Country	N/A	32.2	34.6	33.2	N/A	N/A
n = 117070									
Martinsson et al., 2020	2006-2017	Sweden	Country	N/A	N/A	5.9	94.1	N/A	N/A
n = 16462									
Houttekier et al., 2010	2003	the Netherlands	Country	N/A	4.7	3	90.7	0.7	N/A
n = 30281									
MacNeil-Vroomen et al., 2015	2006	the Netherlands	Country	N/A	3.97	4.24	91.79	N/A	N/A
n = 17814									
Reyniers et al., 2015	2011-2013	the Netherlands	Country	N/A	3.8	1.6	93.1	N/A	1.5
n = 264604									
MacNeil-Vroomen et al., 2021	2012-2017	the Netherlands	Country	N/A	5.82	1.98	90.23	N/A	1.97
n = 81373									
Oosterveld-Vlug et al., 2022	2017	the Netherlands	Country	N/A	10.2	1.8	85.6	N/A	2.5
n = 17148									
Houttekier et al., 2010	2003	Wales, UK	Country	N/A	3.3	52.8	43.8	N/A	N/A
n = 30281									
Reyniers et al., 2015	2011-2013	Wales, UK	Country	N/A	4.2	43.1	48.9	0.1	3.7
n = 264604									
**East Asia and the Pacific (9 studies)**									
Bessho et al., 2005	1992-2002	Japan	City: Kyoto	N/A	18.8	48.3	32.4	N/A	0.6
n = 170									
Nakanishi et al., 2018	1996-2016	Japan	Country	N/A	25	52	23	N/A	N/A
n = 960423									
Koyama et al., 2019	1999	Japan	Country	N/A	27.4	54.8	15.8	N/A	0.56
n = 182077									
Koyama et al., 2019	2000	Japan	Country	N/A	23.3	53.1	18.7	N/A	0.77
n = 182077									
Koyama et al., 2019	2001	Japan	Country	N/A	21.5	55.5	20.6	N/A	0.64
n = 182077									
Koyama et al., 2019	2002	Japan	Country	N/A	20.2	56	21.8	N/A	0.73
n = 182077									
Koyama et al., 2019	2003	Japan	Country	N/A	17.6	57.7	21.1	N/A	0.99
n = 182077									
Koyama et al., 2019	2004	Japan	Country	N/A	15.8	58.9	22.2	N/A	1.39
n = 182077									
Koyama et al., 2019	2005	Japan	Country	N/A	15.8	58.2	24.7	N/A	1.26
n = 182077									
Koyama et al., 2019	2006	Japan	Country	N/A	15.8	58.4	24.2	N/A	11.6
n = 182077									
Koyama et al., 2022	2006	Japan	Country	N/A	12.2	43.6	43.6	N/A	0.6
n = 5572									
Koyama et al., 2019	2007	Japan	Country	N/A	14.3	57.1	26.9	N/A	1.71
n = 182077									
Koyama et al., 2022	2007	Japan	Country	N/A	15.5	42.9	40.2	N/A	1.4
n = 5572									
Koyama et al., 2019	2008	Japan	Country	N/A	14.6	54.9	28.3	N/A	2.24
n = 182077									
Koyama et al., 2022	2008	Japan	Country	N/A	13.5	35.9	47.1	N/A	3.5
n = 5572									
Koyama et al., 2019	2009	Japan	Country	N/A	13	54.5	29.9	N/A	2.58
n = 182077									
Wammes et al., 2021	2009	Japan	Country	N/A	12.8	57.5	19.9	N/A	9.73
n = 149638									
Koyama et al., 2022	2009	Japan	Country	N/A	9.5	35.6	53.5	N/A	1.5
n = 5572									
Wammes et al., 2022	2009-2016	Japan	Country	Pre-reform	23.1	58.2	13	N/A	5.6
n = 216442									
Wammes et al., 2022	2009-2016	Japan	Country	Post-reform	11.6	52.5	24.3	N/A	11.7
n = 216442									
Koyama et al., 2019	2010	Japan	Country	N/A	12.2	51.6	32.8	N/A	3.36
n = 182077									
Wammes et al., 2021	2010	Japan	Country	N/A	12.1	54.9	21.3	N/A	11.8
n = 149638									
Koyama et al., 2022	2010	Japan	Country	N/A	11.7	37.3	47.9	N/A	3.1
n = 5572									
Koyama et al., 2019	2011	Japan	Country	N/A	11.5	50.7	34.3	N/A	3.5
n = 182077									
Wammes et al., 2021	2011	Japan	Country	N/A	11.4	54.1	22.8	N/A	11.8
n = 149638									
Koyama et al., 2022	2011	Japan	Country	N/A	10.3	32.9	54.7	N/A	2.1
n = 5572									
Koyama et al., 2019	2012	Japan	Country	N/A	11.8	49	35.8	N/A	3.47
n = 182077									
Wammes et al., 2021	2012	Japan	Country	N/A	11.7	52.1	23.8	N/A	12.5
n = 149638									
Koyama et al., 2022	2012	Japan	Country	N/A	10	34.1	53	N/A	3
n = 5572									
Koyama et al., 2019	2013	Japan	Country	N/A	11.5	48.3	36.7	N/A	3.48
n = 182077									
Wammes et al., 2021	2013	Japan	Country	N/A	11.5	51.5	25.3	N/A	11.7
n = 149638									
Koyama et al., 2022	2013	Japan	Country	N/A	9.6	30.6	56.7	N/A	3.1
n = 5572									
Koyama et al., 2019	2014	Japan	Country	N/A	10.9	47.4	37.7	N/A	3.92
n = 182077									
Wammes et al., 2021	2014	Japan	Country	N/A	10.9	50.4	26.3	N/A	12.4
n = 149638									
Koyama et al., 2022	2014	Japan	Country	N/A	9.8	31.4	54.9	N/A	3.9
n = 5572									
Koyama et al., 2019	2015	Japan	Country	N/A	10.6	46.4	39	N/A	4.03
n = 182077									
Wammes et al., 2021	2015	Japan	Country	N/A	10.5	49.5	27.1	N/A	12.8
n = 149638									
Koyama et al., 2022	2015	Japan	Country	N/A	9.3	29.2	57.9	N/A	3.6
n = 5572									
Koyama et al., 2019	2016	Japan	Country	N/A	10.5	45	40.6	N/A	3.92
n = 182077									
Wammes et al., 2021	2016	Japan	Country	N/A	10.6	48.1	28.8	N/A	12.5
n = 149638									
Koyama et al., 2022	2016	Japan	Country	N/A	9.4	27.5	59.2	N/A	3.9
n = 5572									
Nakanishi et al., 2023	2018-2021	Japan	Country	N/A	8.6	63.6	25.9	N/A	1.9
n = 653959									
Harada et al., 2024	2019–2023	Japan	Country	N/A	9.9	48.7	38.9	N/A	N/A
n = 279703									
Reyniers et al., 2015	2011-2013	New Zealand	Country	N/A	4.5	14.3	76.6	0.3	4.4
n = 264604									
Reyniers et al., 2015	2007	the epublico f Korea	Country	N/A	20.5	73.6	5.5	N/A	0.4
n = 264604									
**North America (15 studies)**									
Jayaraman & Joseph, 2013	2004-2008	Canada	Providence/State:	N/A	2.6	22	75.2	N/A	0.1
n = 8683			British Columbia						
Quinn et al., 2021	2010-2017	Canada	Providence/State: Ontario	N/A	75	14.1	N/A	N/A	11
n = 14033									
Reyniers et al., 2015	2011-2013	Canada	Country	N/A	3.4	32.3	59.4	N/A	4.9
n = 264604									
Ganguli & Rodriguez, 1999	1987-1996	United States	Providence/State: Washington and Pennsylvania	N/A	12.2	54.6	23.3	N/A	9.9
n = 172									
Lane et al., 1998	1988-1994	United States	Providence/State: South Carolina	N/A	12	34	44	<1	N/A
n = 3109									
Khan et al., 204	1999-2020	United States	Country	N/A	22.4	8.3	57.3	4.1	7.8
n = 1852432									
Teno et al., 2013	2000	United States	Country	N/A	19.9	28.6	45.6	N/A	N/A
n = 126926									
Xu et al., 2020	2000	United States	Country	N/A	12.4	15.6	67.9	N/A	4.1
n = 298453									
Mitchell et al., 2005	2001	United States	Country	N/A	12.8	15.6	66.9	N/A	4.7
n = 88523									
Cross et al., 2020	2003-2017	United States	Country	N/A	18.4	10.1	58.4	4.5	8.6
n = 2778592									
Lo et al., 2023	2004-2021	United States	Country	Ethnicity: White	19.64	9.56	58.12	4.84	7.84
n = 3291422									
Lo et al., 2023	2004-2021	United States	Country	Ethnicity: Japanese	25.91	13.26	41.8	2.77	16.26
n = 3291422									
Lo et al., 2023	2004-2021	United States	Country	Ethnicity: Korean	23.78	17.05	47.36	3.63	8.18
n = 3291422									
Lo et al., 2023	2004-2021	United States	Country	Ethnicity: Asian Indian	43.12	18.25	28.69	4.88	5.06
n = 3291422									
Lo et al., 2023	2004-2021	United States	Country	Ethnicity: Filipino	36.82	19.61	32.68	2.88	8.01
n = 3291422									
Lo et al., 2023	2004-2021	United States	Country	Ethnicity: Chinese	25.95	22.42	40.82	3.56	7.25
n = 3291422									
Lo et al., 2023	2004-2021	United States	Country	Ethnicity: Vietnamese	35.87	23.15	34.74	2.93	3.31
n = 3291422									
Reyniers et al., 2015	2007	United States	Country	N/A	15.3	13.2	62.6	2.9	6
n = 264604									
Teno et al., 2013	2009	United States	Country	N/A	22.8	17.5	48.8	N/A	N/A
n = 126926									
Kim et al., 2024	2010-2019	United States	Country	N/A	77.8	42.6	NA	41.3	N/A
n = 893086									
Xu et al., 2020	2014	United States	Country	N/A	21	9.7	55.6	N/A	13.7
n = 298453									
Nguyen et al., 2022	2015-2020	United States	Country	HBPC^f^	56	12	6	0	26
n = 1148									
Nguyen et al., 2022	2015-2020	United States	Country	Non-HBPC^g^	31	20	16	1	31
n = 1148									
Gotanda et al., 2022	2016-2018	United States	Country	N/A	34.6	16.5	39.6	9.6	N/A
n = 400002									
Chen et al., 2023	Pandemic year 1 (March 2020 – February 2021)	United States	Country	N/A	28.66	13.79	50.92	6.63	N/A
n = 509179									
Chen et al., 2023	Pandemic year 2 (March 2021 – February 2022)	United States	Country	N/A	33.34	13.6	45.19	7.87	N/A
n = 509179									
**Latin America and the Caribbean (one study)**									
Reyniers et al., 2015	2011-2013	Mexico	Country	N/A	69.3	26.2	N/A	N/A	4.5
n = 264604									

**Note:** Data from 48 studies are presented disaggregated by countries

^a^PoD prevalence is presented using a heat map (green = lower proportion; red = higher proportion).

^b^N/A was used for lack of data regarding PoD frequency or associated factors.

^c^Includes only PwD.

^d^Underlying cause of death (UCOD).

^e^Multiple causes of death (MCOD).

^f^Homebased primary care (HBPC).

^g^Non-Homebased primary care (HBPC).

### Trends

A separate analysis was conducted on the eight studies (16.7%) that reported data on PoD for several years to identify trends. Home deaths have presented a constant decrease in Italy [30], Japan [42,43], Germany [25], and Norway [45] since 1999. Conversely, two studies (4.2%) conducted in the United States have shown an increase in home death frequency since 2000 [54,71], with one study (2.1%) showing a spike in 2021 and 2022 [32].

**Fig 2:**
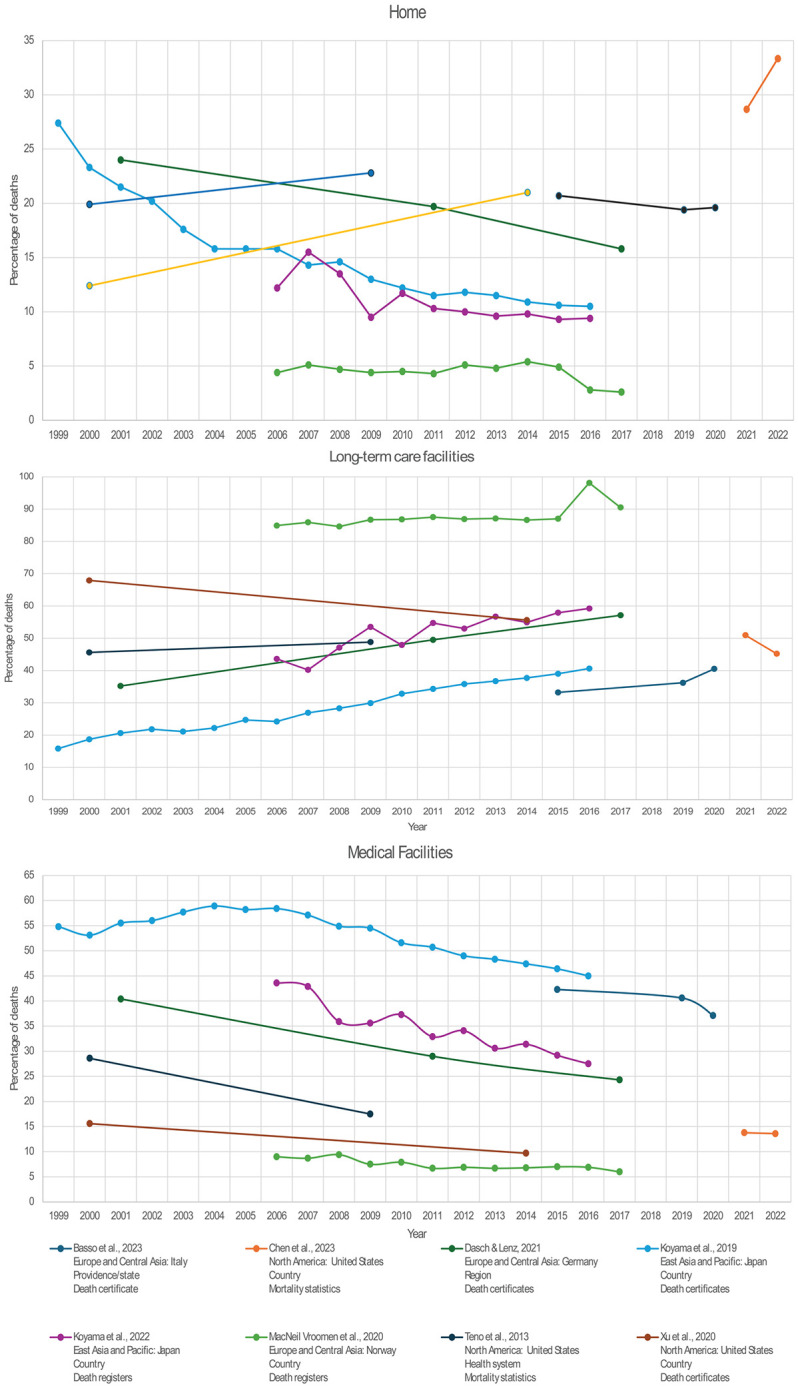
Trends in place of death of people with dementia at home, long-term care facilities, and medical facilities.

Similarly, the results show a decreasing trend in MF deaths over the years, with more pronounced trends in Japan, Italy, and Germany and fewer variations in Norway and the United States. The frequency of deaths in LTCF has risen from 1999 to 2020 in the same countries, except the United States, where one study (2.1%) found an increase in death frequency in LTCF, while two studies, report a decreasing number of deaths in LTCF in the same country [54,71]. Fig 2 shows the change in PoD over the years as shown by these studies.

### Associated factors

**Fig 3:**
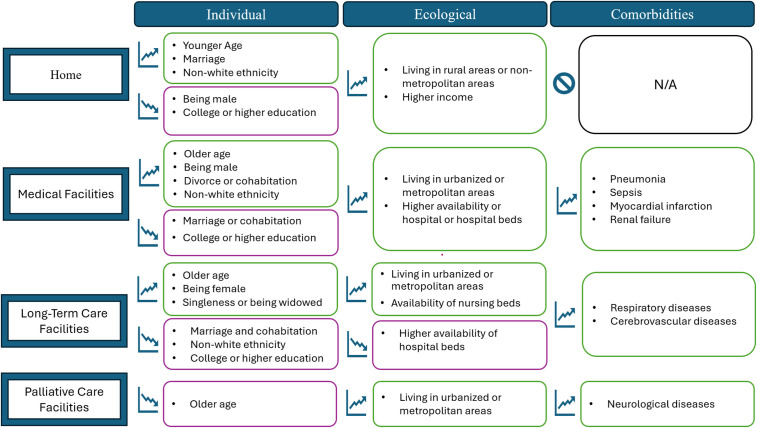
Comorbidities, sociodemographic, and ecological factors associated with the place of death of people with dementia. **Note:** Green-lined boxes contain factors that contribute to increased likelihood, and purple-lined boxes contain factors that contribute to decreased likelihood of dying at a specific PoD.

Nineteen studies (39.6%) reported associations between comorbidities, sociodemographic, and ecological factors and the likelihood of PoD that varied widely in direction and magnitude [13,24,27,28,31,33,37,38,43–45,47,48,51,54,61,62,65,68]. A summary of recurring associations found in these studies is shown in Fig 3.

### Evidence-based suggestion for end-of-life care

Suggestions and recommendations for medical staff, policymakers, facilities’ adaptations, and death planning were retrieved from nine studies [13,24,25,49,58,62,66–68].

Recommendations for the organization of end-of-life care provision included: Enhancing the availability of proper outpatient palliative care for PwD [51,58], increasing investments in care homes [61], promoting access to appropriate end-of-life care [13], and developing integrative services to address care needs across different stages of the disease trajectory [32]. Additionally, the authors suggested that healthcare workers should be able to offer palliative care across all healthcare settings [24,38,49,62,66–68], provide needs-based patient care [24,28,34], and promote proper end-of-life planning [51,58,61].

One single article explored the preferred PoD of PwD [61], reporting that 672 (83.7%) participants included in the study achieved their preferred PoD, and 16.3% stated their preferred PoD but did not achieve it. No other research delved into the preferred PoD.

## Discussion

This scoping review reveals significant variability in the PoD of PwD across different regions and settings. This variability highlights the influence of multiple sociodemographic and contextual factors on end-of-life care and outcomes for PwD that should be further studied.

### Main findings

Reporting the most common PoD of PwD is not possible due to the high variability of the data. In most studies, LTCF were the most frequently reported PoD; however, this trend may be attributed to a representation bias, as most studies (26 out of 48) were conducted with a sample from Europe. Conversely, in East Asia and the Pacific, as well as in North America, MF were found to be the most frequent PoD of PwD. Previous studies suggest that this could be a consequence of social inequality. Countries in North America and East Asia and the Pacific have similar healthcare expenditures to Europe [72]; nevertheless, European citizens face lower inequality levels, greater access to affordable healthcare [73,74], better health-related infrastructure, higher availability of professionals, and better organization and governance [75].

Home was deemed the most frequent PoD in Mexico, which could be explained by the literature’s remarks on higher rates of home deaths in low and middle-income countries (LMIC) [76]. Home was also found to be the most prevalent in the Dumfries and Galloway region in Scotland [60], as well as Italy, and the Andalucía region in Spain [13], which could be a specific phenomenon or cultural factor in the studied region or population, as other results from the same countries [30,37,57,60] do not support these findings. Moreover, the frequency of reported deaths in PCF was minimal across all regions. However, there may be an underestimation of death at PCF, as they may be registered under other labels. Additionally, while the category ‘Elsewhere’ was reported on very few occasions, over one-third of PwD in Hungary die in these locations [13], and its reasons should be further studied.

Most studies were conducted in high-income countries, and only one study presented data from the Latin America and Caribbean region [13]. No study included a population from South Asia, Sub-Saharan Africa, or the Middle East and North Africa. Therefore, results reflect only a partial view of the situation worldwide for PwD, lacking a comprehensive perspective of LMIC, where healthcare systems are usually weaker and where palliative care is in initial phases of development or there are fewer programs for PwD [77,78]. This result is of particular importance, given the rising cases of dementia in these regions and the prevalence of risk factors that health systems have not addressed [2,5].

### Trends

Eight studies (16.7%) provided data from several years, enabling analysis of trends over time [25,30,32,42,43,45,54,69]. Findings showed an overall increase in deaths occurring in LTCF. This may be associated with a parallel rise in LTCF infrastructure development [79,80]. Additionally, studies have found an increased risk of death in LTCF for older individuals and for PwD [81,82].

Home deaths appear to have decreased over time in Italy, Japan, Norway, and Germany, possibly reflecting changes in societal composition (e.g., fewer family members) and the substantial burden families face when managing the demands of PwD [83–85]. Conversely, the home deaths increase in the US, contrary to trends in other regions, is consistent with studies showing that older adults are more likely to die at home than in other PoD in this country [86]. This trend may be attributed to the higher rates of family caregiving [87], healthcare access [73,75], and infrastructure saturation during the pandemic, where most deaths due to COVID-19 occurred in MF, while home deaths increased for all conditions, including dementia [75,88].

### Sociodemographic factors

Overall, our findings reveal that younger PwD are more likely to die at home, whereas older individuals tend to die in institutional settings such as LTCF. Previous studies on PwD support this trend [13,57,81,89]. Contributing factors include the availability of family caregiving [81,87], and the preference for Home as the PoD among older individuals [61,90].

The relationship between education level and PoD was contradictory. Some studies suggested that a higher educational level is linked to a decreased likelihood of dying at home [33,54], while other results indicated a connection between a higher educational level and MF and LTCF death [13]. These mixed findings have also been evidenced in a systematic review with a focus on a worldwide sampling, which found that the relationship between PoD and educational level is inconclusive [91].

Social support and cohabitation are also associated with PoD outcomes. Being married is associated with a higher likelihood of dying at home, whereas being single, widowed, or living in cohabitation is associated with institutional death [33,92]. This finding can be explained by the role of family support in end-of-life care, which decreases the need for external institutions like MF and LTCF [87,91,93,94].

### Contextual/ healthcare factors

Our findings on resource availability and living area suggest that access to available beds, and professionals could influence PoD prevalence. A higher availability of hospital beds increases the likelihood of dying at MF [54], while decreasing the odds of dying at LTCF [92]. Conversely, some findings suggest that a higher availability of nursing home beds increases the chance of dying at LTCF [37,54]. Similar findings have been evidenced, as availability in a specific setting increased the odds of dying at that site, while decreasing the probability of dying at other places [38,93,95–98]. For instance, political decisions to strengthen long-term care facilities clearly increased the likelihood of institutional deaths in the Netherlands [45] and Japan [29,42,50,53].

Additionally, PwD living in urbanized or metropolitan areas are prone to die in MF, LTCF, and PCF. In contrast, those living in rural areas or non-metropolitan areas are more likely to die at home. This variation may be caused by increased access to healthcare and health professionals in urbanized areas, leading to more frequent consultation and, therefore, entry into care institutions [91,93,94].

A higher income was linked with home death, possibly due to better access to outpatient and home-based care [87,90,91,94]. Conversely, lower socioeconomic status has been associated with institutional deaths [91,99,100]. Literature indicates that in low-income countries, access to healthcare and palliative care facilities is limited, as available resources are primarily allocated to medical provision [76,101]. The relationship between income, socioeconomic status, and allocation of resources must be further studied.

### Limitations

This scoping review only included studies with administrative data. Research considering other sources (e.g., caregiver interviews) may provide additional insight into where PwD die, how they do so, and under which conditions. Similarly, administrative data might reflect different variables in each country, and it is known that using death certificates could present inaccurate information [102]. The lack of other types of information limits the exploration of different social and cultural aspects that might be at play regarding PoD.

Furthermore, the lack of quality appraisal in the present scoping review may present as a limitation, as information derived from that process may help to better interpret the charted data. Additionally, the present study excluded gray literature. This could leave out relevant manuscripts from the review that could offer a different perspective on the studied subject or introduce publication bias. Given that dementia tends to be underreported, there may be more PwD who are not considered in the included studies. Moreover, the severity of dementia was not considered in each study, and it may affect the type of care required for PwD approaching the end of life, as well as the PoD [103]. Furthermore, results should be interpreted considering the representation bias, as over 50% of the included studies reported data from Europe, leaving other regions underrepresented.

The wide disparities among the studied factors and PoD sites reported made data synthesis challenging. Although the categories created might partially address PoD differences, specificities from the context of each country or region might not be reflected. For example, LTCFs were more common in Europe but less so in East Asia and the Pacific. Additionally, only a few studies report PCFs as PoD, which could be due to the lack of this type of facility in the country or region, or the lack of PwD dying in them. Additionally, none of the studies included data from participants from Africa, Central and South Asia, or the Middle East. Hence, with this review, we could not delve into specific details of place of care, preferred PoD, or quality of end-of-life care, as well as other factors influencing PoD.

### Implications for future research, policy, and care

This review highlights several gaps in existing literature that future research needs to address. First, studies in LMIC are necessary to provide a more comprehensive overview of the situation of PwD. However, creative approaches that account for common structural challenges are needed. For example, researchers could focus on community-based practices and use qualitative methods to understand where and how PwD live and die, especially in rural or underserved areas. Additionally, research is required on the preferred PoD of PwD and the needs of patients and families, especially in end-of-life care. Achieving the preferred PoD has been identified as essential for improving quality-of-life in terminal care, where actions such as direct inquiry, the reallocation of resources, health care professionals’ training, and the involvement of family can allow people to achieve their preference for PoD [11,104].

Future studies should focus on how the burden of disease affects the quality of life of PwD, and how the site of residence before death relates to PoD. Additionally, research should consider the perspectives of PwD and their families to ensure that care models align with their values and wishes. Moreover, given that only a fraction of the studies looked at variations over the years, future research should further explore changes over time for each region, as contextual factors may vary. Furthermore, as most PoD studies rely on the validity of the data sources retrieved, research is needed on how these sources influence the reliability of studies (e.g., death certificates vs. mortality statistics).

Regarding policy implications, governments and policymakers should prepare for the increasing number of PwD expected in the coming years [2,3] and be ready to evaluate the tailored policies created to respond to the population of each country. For example, given that death at PCF is not common, the integration of palliative care in LTCF, hospitals, and the community into the continuum of dementia care could be widely beneficial [77]; thus, a greater investment in community-based palliative care services is needed to enable more PwD to die at home if they wish to do so.

Additionally, the results of this review suggest that an increase in the availability of beds and the quality of end-of-life care in LTCFs could benefit PwD and their caregivers, as well as that resource accessibility may influence PoD prevalence. Policymakers should consider these relationships to reduce disparities in access to and quality of end-of-life care by ensuring appropriate support and end-of-life care for PwD and their caregivers, regardless of their sociodemographic background.

Last, direct implications for care include planning for a preferred PoD as death approaches [43,61,65] and initiating palliative care promptly to improve end-of-life quality [101]. Moreover, promoting home death and outpatient care should be reinforced as with adequate support, home care can offer better end-of-life quality for the decedent and their family, without facing the possible challenges of care in other settings [51,58,61,89,90].

## Conclusions

Our scoping review reveals that research around dementia and PoD is limited to a few countries/geographical locations, although dementia prevalence is rising. PoD of PwD shows great variations by country, year, and region. These results show the need for social and health policies that ensure access to quality end-of-life care, especially in the most common PoD in each country. Considering the included studies, in Europe, where most data come from, LTCF are the most frequent PoD of PwD, followed by MF. In East Asia and the Pacific, as well as in North America, MF were more common. There is insufficient information for other regions of the world. Some studies also report that the PoD of PwD is influenced by age, gender, marital status, ethnicity, education level, living in urban or rural areas, income level, hospital and nursing home bed availability, and comorbidities. This detailed analysis of the PoD of PwD enables the identification of patterns that may reflect not only individual and cultural preferences but also structural factors such as availability, accessibility, and quality of end-of-life care in each country. These results highlight the need for further research to better understand the conditions surrounding PoD and the place of care of PwD.

## Supporting information

S1 File Preferred Reporting Items for Systematic reviews and Meta-Analyses extension for Scoping Reviews (PRISMA-ScR) Checklist. (DOCX)
